# Treatment of acute respiratory distress syndrome with allogeneic adipose-derived mesenchymal stem cells: a randomized, placebo-controlled pilot study

**DOI:** 10.1186/1465-9921-15-39

**Published:** 2014-04-04

**Authors:** Guoping Zheng, Lanfang Huang, Haijiang Tong, Qiang Shu, Yaoqin Hu, Menghua Ge, Keqin Deng, Liuya Zhang, Bin Zou, Baoli Cheng, Jianguo Xu

**Affiliations:** 1 Shaoxing Second Hospital, 123 Yan An Road, Shaoxing, Zhejiang 312000, China; 2 Children’s Hospital, School of Medicine, Zhejiang University, Zhejiang, China; 3 First Affiliated Hospital, School of Medicine, Zhejiang University, Zhejiang, China

**Keywords:** Mesenchymal stem cells, Adipose-derived, Acute respiratory distress syndrome, Biomarkers

## Abstract

**Background:**

Recent studies have demonstrated that mesenchymal stem cells (MSCs) modulate the immune response and reduce lung injury in animal models. Currently, no clinical studies of the effects of MSCs in acute respiratory distress syndrome (ARDS) exist. The objectives of this study were first to examine the possible adverse events after systemic administration of allogeneic adipose-derived MSCs in ARDS patients and second to determine potential efficacy of MSCs on ARDS.

**Methods:**

Twelve adult patients meeting the Berlin definition of acute respiratory distress syndrome with a PaO_2_/FiO_2_ ratio of < 200 were randomized to receive allogeneic adipose-derived MSCs or placebo in a 1:1 fashion. Patients received one intravenous dose of 1 × 10^6^ cells/kg of body weight or saline. Possible side effects were monitored after treatment. Acute lung injury biomarkers, including IL-6, IL-8 and surfactant protein D (SP-D), were examined to determine the effects of MSCs on lung injury and inflammation.

**Results:**

There were no infusion toxicities or serious adverse events related to MSCs administration and there were no significant differences in the overall number of adverse events between the two groups. Length of hospital stay, ventilator-free days and ICU-free days at day 28 after treatment were similar. There were no changes in biomarkers examined in the placebo group. In the MSCs group, serum SP-D levels at day 5 were significantly lower than those at day 0 (*p* = 0.027) while the changes in IL-8 levels were not significant. The IL-6 levels at day 5 showed a trend towards lower levels as compared with day 0, but this trend was not statistically significant (*p* = 0.06).

**Conclusions:**

Administration of allogeneic adipose-derived MSCs appears to be safe and feasible in the treatment of ARDS. However, the clinical effect with the doses of MSCs used is weak, and further optimization of this strategy will probably be required to reach the goal of reduced alveolar epithelial injury in ARDS.

**Trial registration:**

Clinical trials.gov, NCT01902082

## Introduction

Acute respiratory distress syndrome (ARDS) is a major cause of acute respiratory failure and is often associated with multiple organ failure. Clinical disorders such as pneumonia, sepsis, aspiration of gastric contents, and major trauma can precipitate ARDS. The pathogenesis of ARDS involves lung endothelial injury, alveolar epithelial injury, and the accumulation of protein-rich fluid and cellular debris in the alveolar space [1]. Even with the current advances in lung-protective ventilation and fluid management, patient mortality rate remains high. A clinical diagnosis of ARDS is associated with large financial burdens due to long hospitalization and ICU stays, a poor survival rate, and an increased use of health services after hospital discharge. Most patients who survive an episode of ARDS will sustain some degree of permanent physical disability as well as reduction in their quality of life. In addition, survivors often have long-term neuromuscular, cognitive, and psychological symptoms [2]. To decrease the occurrence of these life-changing consequences, alternative therapeutic options are needed that can reduce lung injury while facilitating and enhancing lung repair.

In the past decade, the preclinical and clinical studies of MSCs have boosted the expectations of both patients and physicians for MSCs as a treatment modality. Unlike embryonic stem cells, the procurement and use of MSCs is less controversial. There are multiple mechanisms responsible for the protective effects of MSCs, including the secretion of multiple paracrine factors capable of modulating the immune response and restoring epithelial and endothelial integrity [3]. Moreover, their immunomodulatory capacity, coupled with low immunogenicity, have opened up possibilities for their allogeneic use, consequently broadening the possibilities for their application. Allogeneic MSCs have been applied to treat graft-versus-host diseases [4], myocardial infarction [5], autoimmune diseases [6], and inflammatory bowel diseases [7]. In May 2012, Canadian health regulators approved Prochymal^TM^, the first allogeneic MSCs-based drug, for acute graft-versus-host diseases in children who have failed to respond to steroid treatment. Bone marrow (BM)-MSCs are the most widely used MSCs in clinical trials. Unfortunately, the harvest of BM is a highly invasive procedure. Furthermore, the number, differentiation potential, and maximal life span of MSCs from BM decline with increasing age [8,9]. Due to their ease of procurement and cell banking, the adipose-derived MSCs have received significant attention over the past few years [10].

Many studies, including publications from our group [11,12], have demonstrated compelling evidence of the benefits of MSCs from both bone marrow [13-15] and adipose tissues [16-18] in animal models for lung injury and ARDS. We hypothesized that allogeneic adipose-derived MSCs serve as a potential therapeutic agent for the treatment of ARDS. In this randomized, placebo-controlled phase I clinical trial, the primary goal was to evaluate the safety and feasibility of systemic administration of allogeneic adipose-derived MSCs in ARDS patients. Secondary goals were to determine potential efficacy and the effect of MSCs on biomarkers for ARDS.

## Materials and methods

### Patient enrollment

This was a single-center, randomized, double-blind, and placebo-controlled study. The study protocol complied with the declaration of Helsinki and was approved by the Research Ethics Committee at Shaoxing Second Hospital (Clinical Trials.gov Identifier: NCT01902082). Written informed consent was obtained from the patient or legally authorized representative before enrolling each patient. Study enrollment occurred between January and April 2013. ARDS was defined and classified according to the Berlin definition [19]. In the new Berlin definition, diagnostic criteria for ARDS rely on 4 categories: (1) timing: within 1 week of a known clinical insult or new or worsening respiratory symptoms; (2) radiography: bilateral opacities – not fully explained by effusions, lobar/lung collapse or nodule; (3) origin of lung edema: respiratory failure not fully explained by cardiac failure or fluid overload, and (4) oxygenation impairment: subdivided into 3 categories according to the degree of hypoxemia severity (mild, moderate and severe). The Berlin definition eliminated the concept of acute lung injury, which now falls in the category of mild ARDS. Eligible patients were at least 18 years of age and diagnosed within 48 hours with a PaO_2_/FiO_2_ ratio of < 200. Exclusion criteria included pre-existing severe disease of any major organs, pregnancy, pulmonary hypertension, malignant disease, human immunodeficiency virus (HIV) infection or if informed consent could not be obtained.

### Study design and treatment

Patients were randomized upon study enrollment. For all patients, a negative fluid balance was maintained by diuretics and fluid restriction. ARDS Network low tidal volume protocol was adopted for standardized ventilator management, targeting a tidal volume of 8 ml/kg of the patient’s ideal body weight and a plateau pressure less than 30 mmHg [20]. Per the requirement of Research Ethics Committee at Shaoxing Second Hospital, frozen MSCs with DMSO and fetal bovine serum were not allowed to be infused to patients directly. For the MSCs group, frozen cells were immediately thawed, cultured with patient’s own serum and harvested in 24-48 hours. Freshly harvested MSCs, at a dose of 1 × 10^6^ cells/kg body weight, were suspended in 100 ml normal saline for peripheral intravenous infusion and administered over 1 hour within 48 hours of enrollment. For the placebo group, a bag of 100 ml normal saline was infused at similar time point. After administration of the MSCs or placebo at day 0, patients were assessed daily at days 1, 3, 5, 7, 14, and 28 (or until hospital discharge or death, whichever occurred first). Patients who were discharged from the hospital before day 28 were asked to return to the study site for assessment. All other aspects of the therapeutic management of the patients were left to the discretion of the clinical team. The primary endpoint was the occurrence of adverse events. Secondary efficacy endpoints included the following: PaO_2_/FiO_2_ ratio, hospital indices (length of hospital stay, ventilator-free days and ICU-free days at day 28), and serum biomarkers of ARDS including IL-6, IL-8 and SP-D.

### MSCs expansion

Normal human adipose-derived MSCs were purchased from ATCC (Cat # PCS-500-011, LOT 59753760, passage 2, Manassas, VA). The donor of the MSCs was a 23 year-old female of Hispanic origin. MSCs were certified to be negative for HIV, HBV, HCV, bacteria, yeast and mycoplasma. After purchase, sterility, viral, and endotoxin tests of the MSCs were performed at the pathology lab of Shaoxing Second Hospital to confirm the certificate of analysis. Cells were then resuspended in expansion media containing Dulbecco’s Modified Eagle’s Medium (DMEM) -low glucose supplemented with penicillin and streptomycin and 2% fetal bovine serum (FBS) (life technologies, Grand Island, NY) plus EGF and FGF (R&D Systems, Minneapolis, MN) at a density of 4000 cells/cm^2^. Cultures were maintained at 37°C in a humidified atmosphere containing 5% CO_2_ in 150 mm dishes (life technologies, Grand Island, NY). When the cultures reached near confluence (>80%), the cells were detached by treatment with trypsin/EDTA and replated at a density of 4000 cells/cm^2^. MSCs were passaged up to a maximum of four times. After sufficient MSCs were expanded, cells were harvested and cryopreserved in 70% culture media, 20% fetal bovine serum and 10% DMSO. Sterility, viral, and endotoxin tests were carried out again after the expansion. Right after each enrollment, 50 ml of peripheral blood was collected and serum harvested from the patients. If a patient was randomized to MSCs treatment, cryopreserved MSCs were immediately thawed, washed with phosphate-buffered saline (PBS), and cultured with the same expansion media above except supplemented with 2% of the patient’s own serum at a density of 15000 cells/cm^2^ for 24-48 hours. Cells were harvested with trypsin/EDTA and quantitated with a hemocytometer. Viral and endotoxin tests were performed prior to the infusion. All cell culture procedures were carried out in good manufacturing practice (GMP) conditions by personnel who had received formal training in GMP within a facility with highly controlled temperature, room air, pressure, etc.

### Characterization of MSC products

Morphology was monitored twice a week throughout the culture period by light microscopy. Immunophenotyping of cultured MSCs was performed using flow cytometry. The following markers were analyzed: CD73, CD90, CD105, CD34, CD45, and human leukocyte antigen (HLA)-DR (BD Biosciences, Franklin Lakes, New Jersey). The samples were analyzed on a FACSCalibur using CellQuest Pro software (BD Biosciences).

For osteogenic differentiation of the expanded MSCs, cells were further cultured with osteogenic medium containing 10% FBS, 0.2 mM L-ascorbic acid 2-phosphate and 0.01 M β-glycerophosphate in DMEM. After 2-3 weeks, the cultures were stained for alkaline phosphatase (ALP) activity. For adipogenic differentiation, MSCs were cultured in adipogenic medium consisting of DMEM and 10% FBS supplemented with 10 μg/ml Insulin, 100 nM dexamethasone, 250 μM isobutylmethylxanthine and 200 μM indomethacin. After 2-3 weeks of differentiation, the cultures were stained with Oil Red O.

### Animal studies

Sixteen C57BL/6 male mice aged 6-8 week-old were randomized into 4 study groups: short term MSCs (2 days), short term placebo, long term MSCs (28 days), and long term placebo. Animal studies were approved by the Institutional Animal Care and Use Committee at Zhejiang University. Mice received one high dose of intravenous infusion of 2 × 10^8^ expanded cells/kg of body weight or normal saline at day 0. Mice were sacrificed at day 2 or day 28. At the sacrifice, serum was harvested for monitoring renal function, liver function, cardiac enzymes, and pancreatic enzymes. Kidney, liver and lung samples were paraffin-fixed for histopathological analysis.

### Cytokine assays

Five milliliters (ml) of peripheral blood were collected from patients immediately before MSCs or saline treatment (day 0) and day 5 after treatment. Serum samples were collected by centrifugation at 1,500 g for 10 minutes and stored at -70°C until assay at the end of the trial. IL-6, IL-8 and SP-D levels were determined by commercial enzyme-linked immunosorbent assays (ELISA) (R&D Systems, Minneapolis, MN).

### Statistical analysis

Continuous variables were expressed as mean ± standard deviation (SD). Comparisons of continuous variables between two groups were performed by using unpaired Student’s *t*-test. Comparisons within groups were performed by using paired *t*-test. Differences were deemed statistically significant at *p* < 0.05.

## Results

### Adipose-derived MSCs

**Figure 1:**
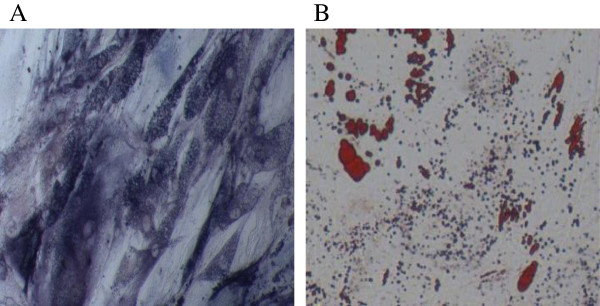
**Differentiation potential of human adipose MSCs. A)** Osteogenic differentiation: cells stained positive for ALP activity; and **B)** Adipogenic differentiation: cells stained positive for Oil Red O. Magnification, 100×.

Adipose-derived MSCs were spindle-shaped with a fibroblast-like morphology and were attached to the plate during cell culture. These characteristics were well preserved during subculture for a total of 4 passages before harvest. For phenotypic characterization of MSCs, surface protein expression at the end of expansion was examined by flow cytometry. The MSCs were positive for CD73 (96.7%), CD90 (97.1%), and CD105 (98.3%), but were negative for CD34 (0.75%), CD45 (1.28%), and HLA-DR (0.73%). The expanded MSCs preserved the abilities of osteogenesis as determined by alkaline phosphatase staining (Figure 1A) and adipogenesis as assayed by Oil Red O staining (Figure 1B).

### Animal studies with MSCs

**Figure 2:**
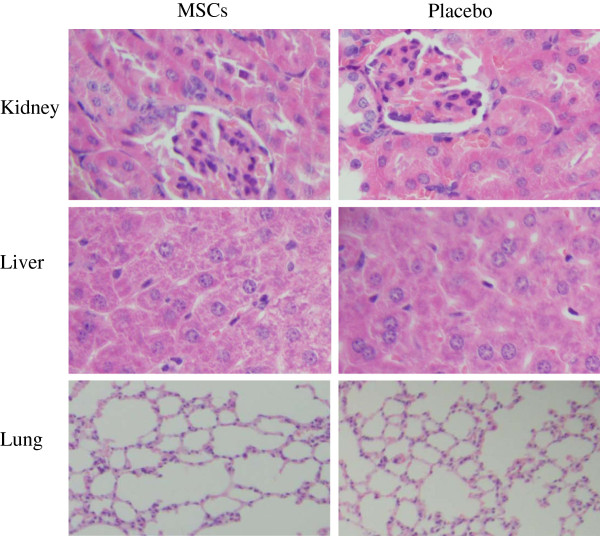
**Histology of kidneys, liver and lungs after MSCs treatment.** Mice were administered intravenously with one dose of 2 × 10^8^ MSCs/kg of body weight or placebo. After 2 days, kidney, liver and lung samples were harvested for H&E staining.

After administering one high dose of 2 × 10^8^ MSCs/kg or normal saline at day 0 via intravenous infusion, no mouse death was observed during the 28-day study period. There were no significant differences in liver (alanine aminotransferase and total bilirubin) and kidney (creatinine and blood urea nitrogen) function between the two groups on both day 2 and day 28 (Table 1). MSCs treatment did not alter cardiac enzymes, pancreatic enzymes and body weight (data not shown). Mice treated with MSCs did not show any histopathological changes in the liver, lungs, or kidneys at both day 2 (Figure 2) and day 28 (data not shown).

**Table 1:** Liver and kidney function by treatment group

		**MSCs group**	**Placebo group**	***p Value***
		**(n = 4)**	**(n = 4)**	
Bilirubin (mg/dl)	Day 2	0.19 ± 0.05	0.16 ± 0.02	0.30
	Day 28	0.20 ± 0.05	0.16 ± 0.03	0.29
ALT (u/l)	Day 2	39.75 ± 7.32	40.50 ± 4.36	0.87
	Day 28	48.50 ± 6.45	61.33 ± 30.89	0.55
Creatinine (mg/dl)	Day 2	0.16 ± 0.01	0.17 ± 0.01	0.39
	Day 28	0.21 ± 0.01	0.24 ± 0.02	0.05
BUN (mg/dl)	Day 2	9.28 ± 1.28	10.62 ± 2.02	0.31
	Day 28	11.64 ± 0.95	11.19 ± 0.54	0.44

Data are presented as mean ± SD. *p* values were calculated using Student’s t-test. Bilirubin = Total bilirubin; ALT = Alanine aminotransferase; BUN = Blood urea nitrogen.

### Patients

A total of 25 ARDS patients were screened for enrollment in the study. Of this number, 13 patients were not enrolled because of the exclusion criteria or refusal to participate the study. The study population is comprised of 6 patients randomized to the MSCs group and 6 patients to the placebo group. Baseline demographics with no statistically significant differences between the study groups are summarized in Table 2.

**Table 2:** Baseline characteristics of the patients

**Characteristic**	**MSCs group**	**Placebo group**	***p *** **value**
	**(n = 6)**	**(n = 6)**	
Age (years)	66.7 ± 20.4	69.8 ± 9.1	0.74
Sex, male/female	6/0	5/1	
Causes of ARDS, n			
Pneumonia	5	5	
Aspiration pneumonitis	1	1	
Baseline APACHE II score	27.2 ± 6.4	23.0 ± 5.1	0.24
Baseline respiratory variables			
PaO2/Fi02	122.4 ± 42.0	103.5 ± 32.2	0.40
Respiratory rate	30.7 ± 3.3	34.0 ± 6.2	0.27
PH	7.42 ± 0.08	7.36 ± 0.24	0.56
Preexisting diseases			
Hypertention	3	3	
Coronary artery disease	1	1	
Neurologic disease	5	3	
Chronic pulmonary disease	1	0	
Diabetes	2	1	

Data are presented as patient number or mean ± SD. *p* values were calculated using Student’s t-test. APACHE = Acute Physiology and Chronic Health Evaluation.

### Adverse events

Within 48 hours of randomization, patients received one dose of 1 × 10^6^ cells/kg body weight or saline as a single intravenous infusion over 60 minutes. Study drugs were well tolerated. No adverse events were recorded during infusions. One patient from each group presented with diarrhea one day after study drug treatment and resolved within 48 hours. One patient in the MSCs group developed rash in the chest area after the infusion and resolved spontaneously over 24 hours. During the study period, one patient in the MSCs group died of multiple organ failure. Deaths occurred in two patients in the placebo group with one multiple organ failure and the other sepsis. None of the deaths were considered to be related to the study drugs by the clinical investigators and were consistent with the patients’ existing disease processes. All the remaining patients completed the 28-day follow-up period. There were no other adverse events or serious adverse events.

### Oxygenation index and outcomes

**Figure 3:**
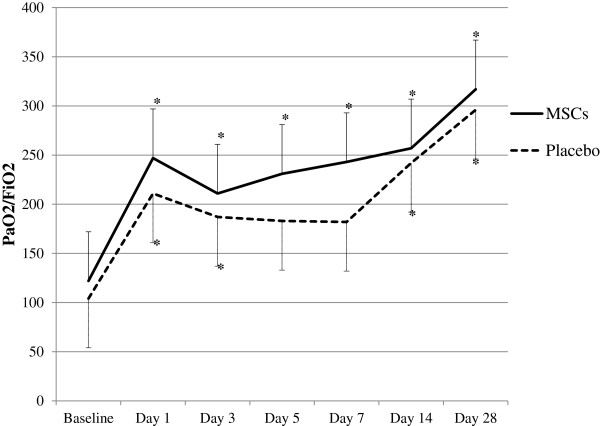
**Time course of PaO**_**2**_**/FiO**_**2 **_**ratio after MSCs or saline treatment.** Values are depicted as mean ± SD. *indicates *p* < 0.05 when compared with the baseline values for that particular study group. No significant differences were found between the two study groups at any of the time points.

As part of the safety and efficacy assessment, an evaluation of the oxygenation index and patient outcomes was conducted. Significant improvements in oxygenation index (PaO2/FiO2) from baseline were observed in all data points in the MSCs group. In the placebo group, there were no significant improvements at days 5 (*p* = 0.05) and 7 (*p* = 0.05) as compared to baseline. The PaO_2_/FiO_2_ did not differ significantly between MSCs and placebo groups at all time points (Figure 3). Assessment of hospital indices did not reveal significant differences in length of hospital stay, ventilator-free days and ICU-free days at day 28 between the two study groups (Table 3).

**Table 3:** Hospital indices by treatment group

	**MSCs group**	**Placebo group**	***p *** **Value**
Days in hospital	33.7 ± 6.3	27.8 ± 5.0	0.10
ICU-free days at study day 28	4.0 ± 8.2	4.3 ± 5.7	0.94
Ventilator-free days at study day 28	11.2 ± 11.5	7.3 ± 7.8	0.52

Data are presented as mean ± SD. *p* values were calculated using Student’s t-test.

### Serum ARDS biomarkers

**Figure 4:**
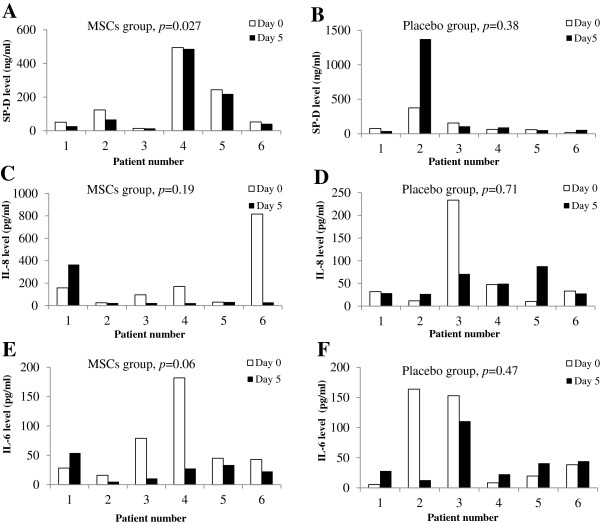
**Paired serum ARDS biomarkers at day 0 and day 5 within MSCs and placebo groups.** SP-D, IL-8 and IL-6 levels at day 0 and day 5 in the MSCs group **(A, C, and E)** and the placebo group **(B, D and F)** were determined by ELISA. Values are the measured concentrations for individual patients. *p*-values within each group between day 0 and day 5 were calculated using paired *t*-test.

There were no statistically significant differences in serum SP-D, IL-6 or IL-8 levels between the MSCs and placebo groups at both day 0 and day 5 (Table 4). In the placebo group, SP-D, IL-6 or IL-8 levels were similar between day 0 and day 5 (Figure 4B, D, F). These findings are in agreement with those reported in other ARDS studies which showed no changes in biomarkers during the first week of ARDS development [21,22]. In the MSCs group, serum SP-D levels at day 5 were significantly lower than those at day 0 (*p* = 0.027) (Figure 4A). The IL-6 levels at day 5 showed a trend towards lower levels as compared with day 0, but this trend was not statistically significant (*p* = 0.06) (Figure 4E). Although the mean value for IL-8 at day 5 was much lower than that of day 0 (Table 4), the difference was not statistically significant due to the variation of the data (*p* = 0.19) (Figure 4C).

**Table 4:** Lung injury biomarkers by treatment group

	**MSCs group**	**Placebo group**	***p *** **Value**
SP-D day 0	163 ± 182	124 ± 131	0.68
SP-D day 5	142 ± 184	285 ± 533	0.55
IL-8 day 0	216 ± 299	61 ± 86	0.25
IL-8 day 5	82 ± 139	49 ± 26	0.57
IL-6 day 0	66 ± 61	65 ± 74	0.99
IL-6 day 5	26 ± 17	43 ± 35	0.29

Data are presented as mean ± SD. SP-D = surfactant protein D (ng/ml); IL-8 = interleukin 8 (pg/ml). IL-6 = interleukin 6 (pg/ml). *p* values were calculated using unpaired Student’s t-test.

## Discussions

Animal studies from our research group and others have showed that MSCs from both bone marrow [11-15] and adipose tissues [16-18] are able to ameliorate lung injury induced by bleomycin and lipopolysaccharide. These studies have laid the foundation for ARDS clinical trials with MSCs. The clinical phase I study reported here was performed to assess the feasibility and safety of allogeneic adipose-derived MSCs in ARDS. The safe use of MSCs was demonstrated by the absence of clinical complications related to cell infusion and short-term adverse events. Matthay et al. is studying the safety of allogeneic BM-MSCs in ARDS (NCT01775774). To our knowledge, our study documents the first clinical trial using adipose-derived MSCs to treat ARDS.

The anti-inflammatory/immunomodulatory effect of MSCs provides a therapeutic rationale for ARDS. It has been reported that the pathogenesis of ARDS involves procoagulant and inflammatory mechanisms as well as damage to the epithelial and endothelial compartments [23]. Biomarkers that reflect inflammation (IL-6, IL-8) [24], coagulation (plasminogen activator inhibitor-1, protein C, thrombomodulin) [25], endothelial cell injury (von Willebrand factor) [26], and epithelial cell injury [SP-D and receptor for advanced glycosylation end products (RAGE)] [27], have all been linked to increased disease severity and poorer clinical outcomes in patients with ARDS. Our results suggest that the MSCs may be effective in decreasing epithelial cell injury as evidenced by reduced SP-D levels at day 5 after MSCs treatment. The levels for pro-inflammatory cytokine IL-6 were decreased with only marginal significance (*p* = 0.06) in MSCs group. Therefore, the present data are not sufficient to support a conclusion that MSCs exert their effects through alleviating lung inflammation.

In the present study, MSCs were administered through peripheral intravenous infusion. Intravenous delivery of MSCs is especially advantageous to lung diseases. Other studies showed that the majority of administered stem cells were initially trapped in the lungs. Infrared imaging revealed stem cells evenly distributed over all lung fields [28]. Systemic administration of MSCs was recently reported in chronic obstructive pulmonary disease (COPD). There were no significant differences in the overall number of adverse events, frequency of COPD exacerbations, or worsening of disease in MSCs-treated patients. Pulmonary function testing and quality of life indicators remained the same after MSCs treatment. For patients who had elevated C-reactive protein levels at study entry, an early significant decrease in the levels of circulating C-reactive protein was demonstrated in MSCs-treated group [29].

Adipogenic precursors were first isolated from human adipose tissue by plastic adherence [30]. Adipose-derived MSCs were identified and characterized in human fat tissue by Zuk et al. in 2001 [31], and this led to the recognition of adipose tissue as an alternative to BM for MSCs. BM-MSCs reside in the bone marrow stroma in relatively small quantities. It has been estimated that they comprise about 0.001%–0.01% of the total marrow nucleated cells [32], whereas the proportion of adipose-derived MSCs is approximately 2% of all nucleated cells of adipose tissue [33]. This difference is particularly relevant for making adipose-derived MSCs more suited for clinical applications due to their ease of accessibility.

Adipose-derived MSCs have other advantages as compared with BM-MSCs. It was initially shown that both BM-MSCs and adipose-derived MSCs exhibit immunosuppressive properties *in vitro*[34]. Adipose-derived MSCs can be more effective suppressors of immune response. They were significantly better than BM-MSCs in inhibiting both the differentiation of blood monocytes into dendritic cells as defined by CD83 expression and the expression of co-stimulatory molecules (CD80, CD86) on the surface of mature monocyte-derived dendritic cells. Adipose-derived MSCs were more powerful than BM-MSCs at stimulating the secretion of immunosuppressive cytokine IL-10 by dendritic cells [35]. It has been demonstrated that adipose-derived MSCs show a significantly greater angiogenic potential compared with BM-MSCs [36], and may be more effective in cardiovascular pathologies associated with ischemia.

Allogeneic BM-MSCs, Prochymal^TM^, has been approved in Canada and New Zealand for acute graft-versus-host diseases in children who have failed to respond to steroid treatment. Allogenic adipose-derived MSCs have been tested to treat several diseases. An open-label, single-arm clinical trial was conducted for Crohn’s disease. Twenty-four patients were administered intralesionally with 20 million adipose-derived MSCs in each draining fistula tract. A subsequent administration of 40 million adipose-derived MSCs was followed if fistula closure was incomplete at week 12. No safety concerns were revealed at 6 months follow-up. At week 24, 69.2% of the patients showed a reduction in the number of draining fistulas with 56.3% of the patients achieving complete closure of the treated fistula [7]. Vanikar et al. administered allogeneic adipose-derived MSCs along with hematopoietic stem cells intraportally in 11 patients with insulin-dependent diabetes and followed the patients for 7 months. Clinical parameters improved significantly as evidenced by a decreased exogenous insulin requirement, reduced levels of glycosylated hemoglobin, elevated serum c-peptide levels, and resolved diabetic ketoacidosis events [37]. Allogenic adipose-derived MSCs have been explored as a salvage therapy of 6 patients with severe steroid-resistant acute graft-versus-host diseases [38]. Complete response was achieved in 5 patients, 4 of them were still alive after a median follow-up of 40 months. All 4 survivors were in good clinical condition and in remission of hematological malignancy [38].

With the application of MSCs in the clinical setting, there is no standard protocol regarding how to expand these cells with GMP. Most existing expansion protocols use DMEM supplemented with FBS. However, FBS is a source of xenogeneic antigens and carries the risk of transmitting animal viruses and prions [39]. Immunological reactions and anti-FBS antibodies have been observed after transplantation in allogeneic hematopoietic stem cell recipients [40]. As an alternative for FBS, platelet lysate [41], both autologous [42] and allogeneic human serum [43], and serum-free medium have been tested for MSCs expansion [44]. To mitigate the allergic reactions in seriously ill ARDS patients and meet the requirements of the Research Ethics Committee at Shaoxing Second Hospital, MSCs were cultured in autologous human serum for 24-48 hours after enrollment in the present study. This delay in MSCs administration may have reduced the effect of MSCs in ARDS.

Our pilot study is limited primarily by the small sample size. The current sample size limits the statistical rigor and power of our findings and, thus, our conclusions regarding safety and efficacy. Another limitation is that the follow-up period was only 28 days. Longer follow-up periods are essential in evaluating the long-term effects of the cells. Finally, our study lacked data regarding the time–response relationship and the dose–response relationship for MSCs. What remains unknown is how often or how many MSCs should be administered in ARDS.

## Conclusions

Due to the small sample size, only limited effects can be observed in this preliminary study. Nevertheless, the findings demonstrated that infusion of allogeneic adipose-derived MSCs was safe and there were no significant adverse events related to the MSCs in ARDS. The change in ARDS biomarker, SP-D, after treatment may suggest the protective effect of MSCs. Additional large studies with a long follow-up period are necessary to confirm the safety and efficacy profile of MSCs in ARDS and to establish the best strategy for their administration, including concomitant medication and dosage.

## Abbreviations

MSCs: Mesenchymal stem cells; ARDS: Acute respiratory distress syndrome; SP-D: Surfactant protein D; BM: Bone marrow; ALT: Alanine aminotransferase; APACHE: Acute physiology and chronic health evaluation; BUN: Blood urea nitrogen; GMP: Good-manufacturing practice; FBS: Fetal bovine serum; PBS: Phosphate-buffered saline; HLA: Human leukocyte antigen; ELISA: Enzyme-linked immunosorbent assay; SD: Standard deviation; RAGE: Receptor for advanced glycosylation end products; COPD: Chronic obstructive pulmonary disease.

## Competing interests

The authors declare that they have no competing interests.

## Authors’ contributions

Conception and design: GZ, LW, QS, JX; Acquisition of Data: GZ, LW, HT, YH, KD, LZ, BZ, BC, JX; Analysis and interpretation: GZ, LW, HT, QS, YH, JX; All authors contributed to drafting the manuscript of important intellectual content and final approval of the manuscript.
